# Embracing the value of research data: Introducing the JCHLA/JABSC Data Sharing Policy

**DOI:** 10.29173/jchla29536

**Published:** 2021-04-02

**Authors:** Kevin Read, Alanna Campbell, Vanessa Kitchin, Heather MacDonald, Sandra McKeown

**Affiliations:** 1 Associate Librarian, University of Saskatchewan, Saskatoon, SK; 2 Public Services Librarian, Northern Ontario School of Medicine, Sudbury, ON; 3 Librarian: Dentistry & Biomedicine, University of British Columbia, Vancouver, BC; 4 Health and Biosciences Librarian, Carleton University, Ottawa, ON; 5 Health Sciences Librarian, Queen’s University, Kingston, ON

## Abstract

Health sciences researchers are being asked to share their data more frequently due to funder policies, journal requirements, or interest from their peers. Health sciences librarians (HSLs) have simultaneously begun to provide support to researchers in this space through training, participating in RDM efforts on research grants, and developing comprehensive data services programs. If supporting researchers' data sharing efforts is a worthwhile investment for HSLs, it is crucial that we practice data sharing in our own research endeavours. Sharing data is a positive step in the right direction, as it can increase the transparency, reliability, and reusability of HSL-related research outputs. Furthermore, being able to identify and connect with researchers in relation to the challenges associated with data sharing can help HSLs empathize with their communities and gain new perspectives on improving support in this area. To that end, the Journal of the Canadian Health Libraries Association / Journal de l’Association des bibliothèques de la santé du Canada (JCHLA/JABSC) has developed a Data Sharing Policy to improve the transparency and reusability of research data underlying the results of its publications. This paper will describe the approach taken to inform and develop this policy.

## Introduction

Data sharing is a concept that has evolved in research communities over the last ten years. The U.S. National Network of Libraries of Medicine defines data sharing as “the practice of making data used for scholarly research available to other investigators” [1], but openly accessible research results may also be of interest to research participants, journalists, educators, or interested members of the public. Sharing research data can lead to the reuse of data to generate new insights and discoveries [2], catalyze collaborations [3], increase the trust of research findings [4], and is associated with an increased publication citation rate [5].

In Canada, the transition to a stronger data sharing culture is on the horizon. Federal granting agencies are strong advocates for making the results of research as accessible as possible because of the potential benefits to research communities but also for society at large. These publicly funded organizations in Canada (the Tri-Agency), “aspire to advance knowledge, avoid research duplication and encourage reuse, maximize research benefits to Canadians and showcase the accomplishments of Canadian researchers” [6].

The Tri-Agency is in the process of developing a research data management (RDM) policy where funded researchers are required to deposit all research data that directly supports their research conclusions in journal publications, pre-prints, and other research outputs, into a data repository, and to make it accessible when possible [7]. More recently, Canada released a Roadmap for Open Science proposing that all research data generated from federally funded departments and agencies be made interoperable, available, and reusable by January 2025 [8]. Finally, Canada has established a national data repository — the Federated Research Data Repository — for the purposes of aggregating, storing, and increasing the discovery of Canadian research data [9].

Canadian health sciences research has also made strides towards data sharing in recent years: prominent Canadian medical journals have established their own data sharing policies [10]; the Montreal Neurological Institute has become the gold standard for sharing research data, tools, and software as soon as it is developed [11]; and the Canadian Open Genetics Repository has served as a key data sharing resource to take full advantage of laboratory data in diagnosing, managing and treating genetic diseases since 2013 [12]. These achievements are cause for celebration, as many researchers have previously identified the lack of access to data generated by others as a major impediment to progress in science [13].

As health sciences researchers have been asked to share their data more frequently due to funder policies, journal requirements, or interest from their peers, health sciences librarians (HSLs) have simultaneously begun to provide support to researchers in this space through training [14,15], participating in RDM efforts on research grants [16,17], and developing comprehensive data services programs [18-22]. If supporting researchers' data sharing efforts is a worthwhile investment for HSLs, it is crucial that we practice data sharing in our own research endeavours. Sharing data is a positive step in the right direction, as it can increase the transparency, reliability, and reusability of HSL-related research outputs. Furthermore, having the ability to identify and connect with researchers in relation to the challenges associated with data sharing can help HSLs empathize with their communities and gain new perspectives on improving support in this area.

To that end, the Journal of the Canadian Health Libraries Association / Journal de l’Association des bibliothèques de la santé du Canada (JCHLA/JABSC) has developed a Data Sharing Policy to improve the transparency and reusability of research data underlying the results of its publications. In addition to introducing the new Data Sharing Policy, this paper describes the approach taken to inform and develop the policy.

## Policy Development Process

A working group (WG) was formed that included members of the JCHLA/JABSC editorial team, and three librarians with expertise in RDM. The WG took a three-pronged approach to gathering information to develop the policy, which included reaching out to authors of previously published JCHLA/JABSC articles for feedback, reviewing existing journal data sharing policies, and holding an open stakeholder consultation webinar with the HSL community to introduce the draft policy and solicit feedback. This section will describe each of these approaches in more detail.

### Author Feedback

The WG first contacted authors of recently published JCHLA/JABSC research articles and program descriptions to gain a better understanding of the data generated for their research and reports, and to listen to any concerns authors may have about the journal’s transition to a data sharing policy. Authors who had published articles in the past two years were contacted and asked to answer the following questions:

1. Would they be willing to share any research data from their publication with the WG?
2. Had they developed any documentation to make the data more understandable or reusable?
3. Did they have thoughts about where they would deposit their data if asked?
4. What information or guidance would be helpful for them to comply with a data sharing policy?
5. Did they have any concerns about JCHLA/JABSC developing a data sharing policy?

The WG received feedback from eleven authors concerning the policy. Based on the feedback received, several themes emerged that helped inform the types of language that would be needed to develop a clear and manageable policy.

### Definition of Data

Almost all respondents (n=9) raised the issue that they would like the JCHLA/JABSC policy to have a clear definition of what is meant by data within the context of library research, and the types of data that should be shared. The need for a definition of data was strengthened by the fact that several authors (n=5) did not think they had collected data, when in fact they had. For example, authors who had published case studies did not think they had data to share because their publication did not constitute original research, even though they reported data within the manuscript. This feedback made it clear that the data sharing policy would require explicit language on the types of data that should be shared, as well as what article types the policy would apply to.

### Support for Institutional or Open Repositories

All respondents mentioned that if they were required to share their data and could choose where it would be shared, they would select either an institutional (n=7) or open (n=4) repository where they could guarantee access. Other (n=3) respondents mentioned that they would like JCHLA/JABSC to recommend several repository options that included both general and discipline-specific platforms. This feedback paved the way for the policy to include options with descriptions for the types of repositories that would be suitable for HSL-specific research data.

### Concern for Sharing Sensitive Data

Some respondents (n=3) expressed concerns that their research data included sensitive information either from their study participants or their institutions, and that if data sharing was mandatory for all JCHLA/JABSC publications they would be unable to comply. The topics of de-identification, anonymization, data retention, and ethics approval were raised alongside these concerns. Understanding the nuances of sensitive data was crucial for developing a policy that would acknowledge these challenges.

### Concern about Time Investment

A small number of respondents (n=2) expressed concern about the amount of time that would be required to comply with the policy in terms of managing the data, making sure it is in a state where it can be reused by others, and receiving an adequate return on investment in terms of getting credit for making data available. JCHLA/JABSC acknowledges these concerns and has attempted to make the policy clear and concise with the lowest burden possible on prospective authors.

### General Support for a Data Sharing Policy

Most respondents (n=8) specifically indicated that they support the development of a data sharing policy. The reasons stated for support include that data sharing is the way of the future, that it is HSLs responsibility to make research data available for the sake of transparency, to avoid duplication of efforts, and to encourage more collaboration within our profession. Only one of the eleven respondents did not support the implementation of a data sharing policy, citing the fact that they would still want to publish off their data and would not want others to have it. They also expressed uncertainty about whether the small nature of HSL research data would be reused by others, and concern about a lack of credit given to data sharing when being considered for tenure and promotion. Similarly, another author, while supporting the policy overall, mentioned that they would share their data but would not want it to be used by others for their own publications. The WG was encouraged to see such strong support from the majority of authors contacted, and while we acknowledge the concerns that were expressed, we stand by the fact that data sharing for the sake of transparency, reuse, and in the spirit of openness is a worthwhile endeavour.

The feedback received from previous JCHLA/JABSC authors was instrumental in helping to shape the final data sharing policy and provided insight on what types of guidance warranted inclusion within the policy documentation.

### Review of Data Sharing Policies and Principles

After the author feedback had been compiled, the WG examined existing journal data sharing policies and data sharing principles from prominent organizations. The journal policies that were reviewed included: PLOS, Nature, Science, Gigascience, and the Journal of the Medical Library Association. Based on author feedback, specific attention was paid to how these policies defined data required for sharing, what they deemed as unacceptable reasons for not sharing data, the types of licensing criteria they encouraged for authors to place on their data before sharing, and the recommended repositories that were suggested. To supplement these policies, the WG also reviewed the data management and sharing principles from the Canadian Tri-Agency, the Association of College & Research Libraries, the Australian National Data Service, and the Wellcome Trust.

The definitions of data varied across policies and principles, with the exception that all journals indicated the article types where the data sharing policy would apply. Furthermore, these resources provided examples of different data types. There was consistency across all journal data sharing policies in terms of the unacceptable reasons for not sharing; these included not sharing because of personal interest, saving data for future publications, and/or the commercial value of the data (e.g., patents). In terms of licensing data, the two types of licenses that were encouraged across all policies were CC0 or at least as permissive as CC-BY. Finally, depending on the type of journal a wide variety of repositories were recommended. Both general purpose (e.g., figshare, Dryad) and discipline-specific (e.g., GenBank) were mentioned in all journal policies, but institutional repositories were omitted.

Using the WG’s initial author feedback as a guide, the information from these policies and principles was used to shape the policy and its accompanying documentation according to JCHLA/JABSC users’ needs.

### Stakeholder Consultation Webinar and Draft Policy for Comment

The WG hosted a webinar for the HSL community on December 2, 2020. The event announcement was distributed on HSL and library data listservs, social media, and the JCHLA/JABSC website. The webinar provided attendees with a complete overview of the draft JCHLA/JABSC Data Sharing Policy, followed by an open question and answer period. Eight-teen stakeholders registered for the webinar and approximately ten stakeholders attended. After the webinar, the WG circulated a draft policy in both English and French via Google Docs using the distribution methods described above and provided stakeholders with three weeks to add their feedback. This section describes the feedback received from both the webinar and the draft policy review period.

### Supporting Authors without RDM or Data Sharing Experience

The most frequent feedback received was that JCHLA/JABSC should provide additional guidance and resources to assist authors in navigating the new data sharing process. This request came frequently from solo librarians working in hospitals or community settings, where they do not have colleagues that can support them in the same way that an academic or public library might.

To that end, JCHLA/JABSC has developed a comprehensive Data Sharing FAQ on the journal’s website to accompany the policy and address the key questions from stakeholders during this phase. These questions included how to prepare data to be shared in a reproducible and reusable way, which version of data to use when sharing data publicly, how to select a repository, and which data specifically should be shared from their study. JCHLA/JABSC will be leveraging the training resources from the Portage Network including their brief guides, primers, and online training modules specific to data sharing [23] as they were developed by national experts and are widely utilized across research communities.

### Author Expectations for Licensing Data

Stakeholders highlighted that the draft policy asked authors to consider applying a license to their data that is as permissive as possible, such as the Creative Commons Attribution License (CC-BY). Reviewers expressed concern that if licensing data in such a permissive way became a requirement, some of their research data would not be able to be shared due to its proprietary and/or sensitive nature. As a result, the WG revised the policy to encourage the adoption of the most permissive license, but not require it.

### Data Sharing and Participant Consent

Finally, several questions arose during the webinar about how authors should navigate the policy if current research projects did not seek consent from participants to share data publicly, particularly if this was not approved in their ethics application. The WG understands that some leeway will be needed for researchers who started their projects before JCHLA/JABSC announced plans to implement a Data Sharing Policy. The WG assured these authors that they can still comply with the policy by providing a data availability statement that indicates the data is restricted, why it is restricted, and how or if the data can be shared through other means.

Learning from JCHLA/JABSC stakeholders was instrumental in developing the appropriate guidance to support authors of the journal in complying with a data sharing policy. We greatly appreciate the candid and supportive contributions from the HSL community. As a result, the WG feels confident that the policy developed is inclusive of the perspectives of the JCHLA/JABSC community at large.

## JCHLA/JABSC Data Sharing Policy

The JCHLA/JABSC Data Sharing Policy asks authors of research articles and program descriptions to make the data associated with their submitted manuscript available in a public repository or as part of the manuscript (e.g., as a supplementary file). Manuscripts are to include a Data Availability Statement (DAS) describing where the supporting data for the article can be found, including hyperlinks to publicly archived datasets that were analyzed or generated during the study. Manuscripts will be required to have a DAS, regardless of whether the data can be made publicly available, whether access to the data are restricted, or whether, in the case of a Program Description, there are no additional data beyond those reported with the manuscript. Full details of the criteria necessary to write a DAS are included in the Data Sharing Policy that is available on the JCHLA/JABSC Editorial Policies webpage.

Exemptions for sharing data will be made in rare cases where de-identified data cannot be shared due to their proprietary or sensitive nature (e.g., Indigenous data subject to the OCAP principles [24,25], confidential financial information from vendors) or when research projects were initiated before 2021 and did not receive consent from participants to share data. Authors are still required to provide a DAS in such cases, explaining why the data cannot be shared.

The JCHLA/JABSC Data Sharing Policy defines data as the materials collected and reported as evidence for the results or outcomes in either a research article or program description. Data formats may include (but are not limited to) spreadsheets, text files, interview recordings or transcripts, images, videos, outputs from statistical software, or computer code or scripts. Authors are encouraged to save their data in open data formats.

Authors are also encouraged to share accompanying documentation of the data (e.g., data dictionaries, codebooks, readme files) to facilitate the understandability and reusability of the data. Measures should be taken to de-identify data to protect the identity of research participants (see the Data Sharing FAQ page on the JCHLA/JABSC website for guidance).

The JCHLA/JABSC Data Sharing Policy provides a list of recommended repositories where authors can share their data and provides guidance to help authors decide about where best to share. Additionally, guidance on how to choose a license to apply to research data has also been included.

## Timeline for Implementation

Beginning April 1, 2021, the JCHLA/JABSC Data Sharing Policy will undergo a one-year transitional period during which data sharing and data availability statements will be encouraged for new submissions but not required. After the one-year transitional period, starting April 2, 2022, new submissions for research articles and program descriptions will require a DAS within the manuscript with the expectation that research data underlying the results will be made openly available unless it meets the requirements for exemption outlined within the policy.

At the time of submission, the authors’ DAS will be reviewed by a member of the JCHLA/JABSC editorial board. If the availability of data does not meet the requirements described in the policy, the editorial board member will return the manuscript to the author(s) for revision. The article will not be sent out for peer review unless the availability of data is acceptable. Reviewers may also suggest revisions to the availability of data as outlined in the DAS during the peer review process that must be addressed by the author(s). Authors’ data will not be reviewed as part of the peer review process; however, reviewers may request access to data that they consider necessary to evaluate the manuscript.

Authors can choose to embargo their data from the public until their manuscript is accepted for publication, however they must make data available to peer reviewers if it is requested to evaluate the manuscript. If the manuscript is accepted, a member of the JCHLA/JABSC editorial board will notify the authors that their data must be made available as stated in their DAS. The article will not move to production until it includes a complete DAS with active links to the repository, location of documentation necessary to understand the data, and accurate instructions on gaining access to the data.

The JCHLA/JABSC Data Sharing Policy is available on the Editorial Policies page of the journal website and a separate Data Sharing FAQ is also provided.

## Conclusion

The JCHLA/JABSC Data Sharing Policy is a step towards improving the transparency, reproducibility, and reusability of HSL research. As the Canadian data sharing landscape continues to shift towards being more open and collaborative in the health sciences, it is vital that HSLs become proficient in this space. By implementing this policy, we believe that both the HSL research landscape will evolve to be more open and inclusive, and the HSL workforce will be able to transfer the benefits of our newfound data sharing experience to the communities that we support. JCHLA/JABSC is proud of the quality of authors’ work and we look forward to seeing the evolution of HSL research as it becomes increasingly open, transparent, and reusable under this new policy.

## References

[1] National Network of Libraries of Medicine. Data sharing [Internet]. NNLM Thesaurus. [cited 2020 Dec 5]. Available from: https://nnlm.gov/data/thesaurus/data-sharing

[2] Dumontier M, Wesley K. Advancing discovery science with FAIR data stewardship: findable, accessible, interoperable, reusable. Ser Libr. 2018;74(1–4):39–48.

[3] Boland MR, Karczewski KJ, Tatonetti NP. Ten simple rules to enable multi-site collaborations through data sharing. PLOS Comput Biol. 2017 Jan 19;13(1):e1005278. 28103227 10.1371/journal.pcbi.1005278 PMC5245793

[4] Popkin G. Data sharing and how it can benefit your scientific career. Nature. 2019;569(7756):445–445. 31081499 10.1038/d41586-019-01506-x

[5] Piwowar HA, Day RS, Fridsma DB. Sharing detailed research data is associated with increased citation rate. PLOS ONE. 2007 Mar 21;2(3):e308. 17375194 10.1371/journal.pone.0000308 PMC1817752

[6] Government of Canada. Tri-Agency statement of principles on digital data management [Internet]. Government of Canada Policies and Guidelines. 2016. Available from: http://science.gc.ca/eic/site/063.nsf/eng/h_83F_7624E.html

[7] Government of Canada. Draft Tri-Agency research data management policy for consultation [Internet]. Government of Canada Policies and Guidelines. 2018. Available from: http://science.gc.ca/eic/site/063.nsf/eng/h_97610.html

[8] Roadmap for Open Science February 2020 [Internet]. Office of the Chief Science Advisor of Canada; 2020 p. 1–12. Available from: http://science.gc.ca/eic/site/063.nsf/eng/h_979_92.html

[9] Wilson L. Exploring the Canadian Federated Research Data Repositoryservice. Biodivers Inf Sci Stand. 2017;

[10] Kelsall D. New CMAJ policy on sharing study data. CMAJ Can Med Assoc J. 2017;189(34):E1082–E1082. 28847778 10.1503/cmaj.170935 PMC5573541

[11] Owens B. DATA SHARING. Montreal institute going ‘open’ to accelerate science. Science. 2016;351(6271):329–329. 26797995 10.1126/science.351.6271.329

[12] Lerner-Ellis J, Wang M, White S, Lebo MS, Canadian Open Genetics Repository G. Canadian Open Genetics Repository (COGR): a unified clinical genomics database as a community resource for standardising and sharing genetic interpretations. J Med Genet. 2015;52(7):438–45. 25904639 10.1136/jmedgenet-2014-102933 PMC4501169

[13] Fecher B, Friesike S, Hebing M. What drives academic data sharing? PLOS ONE. 2015 Feb 25;10(2):e0118053. 25714752 10.1371/journal.pone.0118053 PMC4340811

[14] Surkis A, LaPolla FWZ, Contaxis N, Read KB. Data day to aay: building a community of expertise to address data skills gaps in an academic medical center. J Med Libr Assoc JMLA. 2017 Apr;105(2):185–91. 28377684 10.5195/jmla.2017.35 PMC5370612

[15] Piorun M, Kafel D, Leger-Hornby T, Najafi S, Martin E, Colombo P, et al. Teaching research data management: an Undergraduate/Graduate Curriculum. J EScience Librariansh. 2012;46–50.

[16] Martin ER. Shaping opportunities for the new health sciences librarian. J Med Libr Assoc. 2013 Oct;101(4):252–3. 24163594 10.3163/1536-5050.101.4.004 PMC3794678

[17] Whipple E, Odell J, Ralston R, Liu G. When informationists get involved: the CHICA-GIS project. J EScience Librariansh. 2013;2(1):41–5. 10.7191/jeslib.2013.1035 PMC3991552 24749002

[18] Courneya J-P, Mayo A. High-performance computing service for bioinformatics and data science. J Med Libr Assoc JMLA. 2018 Oct;106(4):494–5. 30271293 10.5195/jmla.2018.512 PMC6148605

[19] Bardyn T, Patridge E, Moore M, Koh J, et al. Health sciences libraries advancing collaborative clinical research data management in universities. J EScience Librariansh. 2018 Jul 20;7(2):e1130. 30197832 10.7191/jeslib.2018.1130 PMC6124496

[20] Kerby EE. Research data services in veterinary medicine libraries. J Med Libr Assoc JMLA. 2016 Oct;104(4):305–8. 27822153 10.3163/1536-5050.104.4.010 PMC5079493

[21] Read KB, Koos J, Miller RS, Miller CF, Phillips GA, Scheinfeld L, et al. A model for initiating research data management services at academic libraries. J Med Libr Assoc JMLA. 2019 Jul;107(3):432–41. 31258450 10.5195/jmla.2019.545 PMC6579580

[22] Vela K, Shin N. Establishing a research data management service on a health sciences campus. J EScience Librariansh. 2019 Mar 21;8(1):e1146.

[23] Training Resources [Internet]. Portage Network. 2018 [cited 2021 Jan 29]. Available from: https://portagenetwork.ca/tools-and-resources/training-resources/

[24] OCAP® and Information Governance [Internet]. The First Nations Information Governance Centre. [cited 2021 Jan 29]. Available from: https://fnigc.ca/what-we-do/ocap-and-information-governance/

[25] Read K, Donald GM, Mackay B, Barsky E. A Commitment to First Nations data governance: a Primer for Health Librarians. JCHLA. 2014;35:5.

